# Supplementary material for: Vibrio splendidus AJ01 Promotes Pathogenicity via L-Glutamic Acid
Source: Microorganisms. 2023 Sep 17;11(9):2333. doi: 10.3390/microorganisms11092333 (PMC10535082; doi:10.3390/microorganisms11092333)
Supplement: Supplementary file 1 [file microorganisms-11-02333-s001.zip › microorganisms-2560768-supplementary.pdf]

>*vsm*

ATGAACCAACAACGTCAATTAAGCTGGAAAATAGCAGCTATTCTAGGTACATCTTTTGGCTT
TACTGCACACGCTGCAGAGATGGTCAGAGTCGATAATGATGCATTACTACAACAAAGCCTC
GTCGCGCAGTCGAAGAGTGTTGCTCCACTTGAATTAGGTTTTTCTGAAGTTAAACGGGTGG
TATTACCCAATGGAAAAACGAAAGTCCGCTACCAACAAACTCACTTAGGTTTACCCGTCTTT
GATACTACGGTCGTGGCCACCCTCTCCAAGAACCAACCCACTCAGGTGTTCGGGTCAATGG
CACAAGGCATCAGTGGTGACCTATCCAGCATCGCGCCAAAGCTGAATCAGGAACAAGCGAT
TGAAGCCGCACTGTCCGCTCATCGCACGTTTACCGTCGGCAAAAAGTCCATTGAAAATAAA
AATGCCAAACTGATGGTGAGATTGGATGAGAACCAAGTTGCCCAAGTGGTGTATCTGGTCG
ATTTCTTTATCGCGTCCTCAATACCAGAGCGCCCTTTCTACTTTATTGATGCTACGACTGGCG
ATGTTCTCCAAAAATGGAATGGGTTAAACCACGCTAAAGCATTAGGTACTGGCCCAGGTGG
CAACCTCAAAACCACTCGATATGAATATGGCAGTGACTTCCCTAGCTTTCCCATCGACAAAA
CAGGCACGACATGTAAGTTAGAAAACGATTCGGTTAAAACCGTCGATTTGAGGAACGGAA
CGTCCGGCAGCGCAGCCTACAGTTACAACTGTGCTGATGGAACCAACTACACCGACCATAA
GTACATTAACGGGGCTTACTCGCCTCTTAATGATGCGCACTACTTCGGTAATGTCGTATTTGA
TATGTACAAAGAGTGGATGAATACGTCGCCTTTAACTTTCCAGCTGACGATGCGAGTTCATT
ACGGCACGGATTATGAAAATGCCTTCTGGAATGGTACATCCATGACTTTTGGAGATGGCAAA
AATACCTTTTACCCATTAGTCGATATTAACGTGAGTGCTCACGAAGTCAGCCACGGGTTCAC
AGAGCAAAACTCCGGTCTTGTGTACCAAAATATGTCGGGCGGTATGAATGAAGCCTTCTCA
GACATTGCGGGCGAAGCGGCTGAATACTACTTGCGTGGTAATGTGGATTGGGTGGTCGGTA
GCGATATATTCAAATCTGAAGGTGGCTTGCGTTACTTTGATCAACCTTCAAAAGATGGCCGC
TCGATTGACCATGCCTCTCAATACTACGATGGTTTGAACGTTCACTTGTCTAGCGGTGTTTAT
AACCGCGCCTTTTACCTTTTAGCGAACAAGTCTGGCTGGGATGTGCGTAAGGGCTTTGAAAT
ATTCACAGTCGCAAACCAATTGTATTGGACGGCAAACAGTACCTTTGATGCCGGAGCTTGT
GGCGTAGCGAAAGCCGCAGCAGACATGGGCTATGTGGTTGCCGATGTTGAAGATGCTTTTA
ATACGGTAGGCGTTAATGCAAGCTGTGGTGTAACGCCTCCAACTGGTAATGTGCTGACGAA
AGGCACACCGATTGCGAACCTAAGCGGGAATCAATCTTCAGAGAACTTCTACACGTTCACC
GTAGATTCTGTATCAAGCGTGACGGTTTCAATGTCTGGCGGTTCAGGCGATGCCGACCTTTA
CGTTAAATCGGGAAGCAAGCCAACGACATCAAGTTACGACTGTCGCCCTTATCGTGCTGGA
AACAATGAGCAGTGCAGTGTAAGCGCTCAACCGGGCGTGACTTATCATGTGTTACTCCGAG
GGTACTCTAACTATTCTGGCCTAACGTTACGTTTAGATTGA

>*vspC*

ATGCTTGCCAGTGTGTCCAGTTTCTCTTTTGCTCAAAATCAGTGTGAGGTGCTTGATCTACA
ACAGGCGTCCGATCTTGCACTCGCTGTCTCTTCGGCAGGCAACCGTTGCTACAGTTCTTGG
TTCTCTGCATCGGATAATTCACTAGATAATATCTATAGCGAAGCGAGCCTAAGCCGTATTCAA
GTGGCGCTGAACTTAGAAATAGCCCGTTATCGAGGTGAAGCTGAACAAGCACGTAAACTTG
AAAACCTTGGTGAGTTTGTTCGCGCTGCTTATTACGTTCGCTATAACGCGCAAACACAGGAT
TTCTCTGAGGCGTTAAGCCAGCGATTTGCTCAATCTACCAATGCTTTTCTAGCGACCCCTAAT
GCGCAGGACCAAGGACGAGAACAAGTCGGGGCAATGAAAAGTCTGACGTTGATGGTCGAT
AACGTTAAGCAACTTCCACTCACTATGGATGCGCAACTTGCGGCACTTAGACACTTTAATCA
AGAAACGGCGCAAGACTCACAATGGGTCGACGGGCTAAATAATCTGTTCCGAGCAATGGC
AGGACATGCAGCCCGAGATGACTTCTACGATTACATGGCCAGTCATACTCAACATATCGATA
CGCTAGCGGCATTCGCTCGTGATAATACGTGGGCTTTAGATACTGATGCGAGCTTCCTTGTTT

ATAACGCAGTGCGTGAAACTGGTCGTTTTCTGGCAAGCCCAGATAAAGCCACCAAAGATAA
AGCACTGCAAGTTATGCAGCAGGTGATGGTGCAAAACCCATTGGGCAGCGAGCACGATAA
GCTATGGTTAGCGGCGGTCGAGATGATGAGTTACTTTGCGCCAGAAGGCTTAAATGGGTTA
GATCTCGAGCAAGCAAAACGTGATTTAGCAACGCGTGTACTTCCCAATCGTCATGAGTGCC
AAGGGCCTGCGATTATTCGCTCACAAGATTTAACGCAAGCACAAGCGATGGAAGCTTGTAA
TGTGTTGGCCGCCAAAGAGGCCGATTTCCACCAAGTCGCGAACACGGGTCAACAACCTGT
CGCCGACGATAACAATAACCGAGTTGAAGTGGCGGTGTTCGGCAGCAAAGGCAGTTATATC
GATTACTCGTCGTTCTTATTTGGTAATACTACCGATAACGGCGGACAGTACTTAGAAGGCAA
CCCTGCAGAGGTGGGGAACGTGGCGCGTTTCGTGGCTTACCGTTACGCCAATGGCGATGAC
CTCTCTATTCTCAACTTAGAACATGAATACACTCATTATCTCGATGCGCGCTTTAACCAATAT
GGCTCCTTTAGCGACAACTTGGCTCATGGTTATGTGGTGTGGTGGTTAGAAGGTTTTGCCGA
ATTCATGCATTACAAGCAAGGCTACGATGCTGCGGTAAGCTTGATTAACAGCGGGAAGATG
AGCCTGTCGGATGTGCTTGCAACCACTTACTCCCATGATGCTAATCGTATCTACCGATGGGG
CTACTTAGCGGTACGCTTTATGATGGAAGAACATCCTCAAGAGGTGCAGAGCTTGTTGGCA
CTGTCTCGTACTGGCCAATTTTCCAAATGGGCGCAACAGGTGAAAGTGCTTGGCCAGCAAT
ACAATGGTGAGTTTGAGCGTTGGTTAGACACCGTTTCAAGTGAACCAACAGAGCCTACTGA
TCCCGCAGATCCGATTGACCCTACCAATCCGACAGACCAAGCCATGGTACTTACAGCAAAC
CAAAGTGTGGTGTTGAACGGCAAGGCGTACAGCGAGCAGTTGTTCTACGTCGATGTACCAG
AGAACACTAGGCATTTTGAGGTTTCGATTGAAGGAGAAGTTCAAGGTGATGCTGATCTTTA
CATGAGCTACAACAAACAAGCACATTACTATGACTTTGAATTCAGCCAATATCACAATGGCA
GTAATGAGATGGTGACGTTTGAGCCAGAGGCATCAGGCTACATTAAACCGGGTCGCTACTA
CATGAGTATTGCAGGGCGCACCGATTTCAATGACGTGACACTGTTAGCATCGCTAGAGACA
GAAACACCGACACCTCCATCACAAGAACAAGACGACTTAACGCCCGTAGTATTGGAATCCG
GTCAGGATCAAGTATTGACTGTGCATCAACGACGTTATGCAGCAGTCTATGTGCCAGAGGG
CGTACAAGAGGTGCGAGTGTGGTTGAACGATAAGAGTGACAATGCGGAGAATGGACAGTC
GAATACAGGTAATGTTGATCTGTTTGCAAGCCATTCATATTGGCCAACAGCAGGACAACATG
ACTATGCATCGGATTATCGAGGCAGTAATGAATACCTACAAATTCCTGTAACAGAAGAAGGC
TATTTGCACTTCTTACTCAGTGCAGAACAGCAAGGGGATGATGTCGAAATGTTGGTTTATTT
TCACTAG

>*fliC*

ATGCTGAACCAATCTTTGGAGCGCTTAGCTTCAGGGAGTCGTATTAATAGTGCAAAAGACGA
TGCGGCTGGCTTACAAATTTCGAATCGACTGGAAGCGCAGATGAGCGGCATTGATGTCGCG
GTTCGGAACGCTAACGACGGAATCTCCATTATGCAGACTGCAGAAGGGGCGATGAACGAA
ACCACCAATATCATGCAACGCATGCGAGATTTGTCACTGCAAGCGAGTAATGGTTCGAATAG
TCAGTCAGAAAGGACGGCGATTCAAGAAGAAGTGACAGCTCTAAATGATGAGTTGAATCG
AATTGCTGAAACCACTTCATTTGGCGGTAAAAAACTTCTTAACGGTAGTTTTGGTAGCTCAT
CATTTCAAATCGGTGGCAGTTCTGGTGAAGCGGTGCAGATAGGCTTGAAAAATATGCGCAC
CGATGATATCAACATGGGGGGCTTTAGCTATGTCGCTAATGGCATGGCCAGCGACTCGTGGG
AAGTCAAACCCGACCAGAACGATATTACGATGTCGTTCACCGACCGTTTTGGTCAGCCACA
AGAGATCACCATCAACGCGAAAGCGGGCGATGATATTGAAGAGTTGGCGACTTACATTAAT
GGTCAAACGGATCTGGTCTCGGCTTCTGTCAACGATGAAGGGCAACTTCAGATCTATATGTC
AGGTGAAGACACGGCAGGCACGATCTCTTTCTCAGGTTCGTTAGCCAGTGAGCTTTCGATG
TCGGTGGGTTATTACGAATCAGTCGATGACATCAATGTGACCGATGTGGGTGGAGCGCAGC

GTGCTGTCTCTATTTTGGACACCGCGATGAAGTATGTAGACAGTCATCGATCCGAATTAGGC
GCGATGCAAAACCGCTTTGACCATGCAATAAACAATCTCGAAAACGTTCATGAGAACTTGG
CGACATCCAACAGCCGAATCAAAGATACCGACTACGCCAAAGAAACTACTCAAATGCTCAA
GCAGCAAATACTGCAGCAAGTGAGTACCACAATATTGGCTCAAGCAAAGCAAGCGCCGAA
TCTTGCCTTAACCTTACTGGGTTAA

>*hop*
ATGGAACTGGAAACTTTACTAAACACGCTGACGCAAGCCCCAGAAAGCATTCAATTTGAAG
ATACGATGCAGGTGATCGAGGCGAACTATGACTTTTCTGAGAGTGAGTTTCGCAATGGTGAT
GTAGTTAATGTGGCGGGACAGAACAATGGTTCGTGTAAGATTTTTGCTTTTGGTTTAGCTCA
AGATCTTTCTCCCGAGCAAACACTGGCTTGTTTTGGGCAGTTTTATCGCAACGATGTATTAG
GTTTTCCTGAGAATACCGACCATCAAAACATTCGTAATTTTATGATTCATGGTTGGAGTGGTG
TACAGTTTTCTCAGCCTGCGCTGACGGTAAAAGCGAAGTAA

>*vshppd*
ATGGTGGATACATACAACCCGCTTGGTACGGATGGATTCGAGTTTGTTGAATACACAGCTGT
CGACCACAAGGGAATTGAGCAACTTAAAGCGCTGTTTGTGTCACTTGGTTTTGCTGAGATC
GCCAAGCACCGTTCAAAAGAGGCTTGGCTGTATCGACAAGGTGACATTAACTTTATCGTCA
ATGAACAACCACACAGCCAAGCGGAAGCGTTCGCTAAAGTGCATGGTCCATCGGTGTGTG
GCATGGCTTTTCGTGTCAACGAAGCAACCGCTGCTATGGATCAAGCGTTCAAGGGCGGTGG
TGAAGAGTACAAGACAGAAATCGGGCCGATGGAGCTGAGTATTCCTGCCATTTACGGTATC
GGTGAAAGCCTGCTCTATTTTGTGGATCGCTATGGTAAGCAGAGTATCTATGACGTCGATTTT
CGATTCTACGATGATGCAGAGGAGCGCATGGCCGAAGCCAATGTTGGCTTGTATGAAATTG
ACCATCTGACGCACAACGTTAAGCAAGGCAACATGGATGTGTGGTCTGGGTTCTATGAGCG
TCTTGGTAACTTCCGTGAGATTCGATACTTCGATATTGAAGGTAAGCTGACAGGCCTTGTGA
GCCGTGCAATGACCTCCCCATGTGGCAAGATTCGAATCCCAATCAATGAGTCTTCAGATGAC
AAATCACAAATTGAAGAGTTCATCCGCGAATACAACGGCGAAGGCATTCAACACATCGCGC
TTGCAACGGATGACATCTACAAAACCGTCAAAACCCTGCGTGATCGCGGCATGGACTTTAT
GCCAACTCCTGATACTTATTACGAGAAAGTTGACGATCGTGTGAAAGGCCACGGAGAAGAC
ACTGATCTGCTGCGCGAGCTACGCGTTCTGATTGATGGTGCGCCGACCAAAGACGGCATCT
TACTGCAAATTTTCACGCAGACCGTAATCGGGCCTGTGTTCTTTGAAATCATTCAGCGTAAA
GGCAACGAAGGCTTTGGCGAAGGTAACTTCAAGGCGTTGTTCGAATCGATTGAAGAAGAC
CAGATTCGTCGAGGAGTATTAGACGATGCATAA
